# Medium-Chain Triglyceride (MCT) Content of Adult Enteral Tube Feeding Formulas and Clinical Outcomes. A Systematic Review

**DOI:** 10.3389/fnut.2021.697529

**Published:** 2021-08-02

**Authors:** Maurizio Muscaritoli, Lorenzo Pradelli

**Affiliations:** ^1^Department of Translational and Precision Medicine, Sapienza University of Rome, Rome, Italy; ^2^AdRes-Health Economics and Outcome Research, Turin, Italy

**Keywords:** lipid composition, medium-chain triglycerides, MCT, clinical outcomes, enteral nutrition: enteral tube feeding formulas

## Abstract

Available data on the effect of lipid composition of enteral formulas on clinical outcomes are controversial. The present systematic review was performed in order to verify whether the presence of specific lipidic substrates, namely medium-chain triglycerides (MCT), in enteral tube feeding formulas is associated to measurable clinical benefits in patients receiving enteral nutrition in different clinical settings, including home enteral nutrition (HEN). The results of this systematic review highlight a lack of robust evidence supporting the use of specific types of lipids in standard or disease-specific formulas. Evidence exists, however, that MCT-containing formulas are safe and well-tolerated. Further, well-designed, adequately powered, randomized controlled trials would be needed in order to assess the superiority of MCT- containing enteral formulas over other standard or disease-specific commercially available enteral products.

## Introduction

Enteral nutrition, or enteral tube feeding, is defined as nutrition therapy given via tube or stoma into the intestinal tract distal to the oral cavity. Enteral formulas used with this technique are defined as Foods for Special Medical Purposes (FSMP), as established by the Regulation (EU) n° 609/2013 of the European Parliament and of the council (1, 2).

Guidelines refer to standard enteral formulas when their composition is developed to reflect the reference values for macro- and micronutrients for a healthy population, mimicking as close as possible the normal nutritional intake. Indeed, these kinds of products usually contain whole protein, lipid and a source of carbohydrates and fibers (3).

Depending on clinical indications, macronutrient composition of enteral nutrition formulas may vary with respect to carbohydrate, protein and lipid composition.

Carbohydrates may be present in the form of polysaccharides, oligosaccharides (mainly), maltodextrins, sucrose, fructose and glucose from different starches, including corn, and tapioca. Soluble and/or insoluble fibers may also be present (4).

The protein moiety of enteral feeding formulas may be represented by whole or hydrolysate milk proteins (either casein, whey, or both) or small peptides, mainly di- and tri-peptides (4).

In most enteral formulas, lipids are mainly, or exclusively present in the form of corn or soybean oil-derived long-chain triglycerides (LCT) which ensure delivery of adequate amounts of essential fatty acids (EFA), but other lipid substrates may also be present, such as mono-unsaturated fatty acids (MUFA) from safflower and canola oils. Some disease-specific enteral feeding formulas are enriched with gamma-linolenic acid (GLA) and/or omega-3 fatty acids, namely the docosaexahenoic (DHA) and eicosapentaenoic (EPA) acid. With the sole exception of a formula obtained by blenderized natural food, enteral feeding products do not contain cholesterol, while they may contain animal-derived lipids in the form of fish-oil derived omega-3 fatty acids. Lipids in enteral formulas play a major nutritional role, since increasing their relative amount may significantly contribute to the caloric density of the formula, with minimal impact on osmolarity and osmolality, thus allowing to reach daily caloric target within smaller delivered volumes. Besides their role as an energy source, however, some lipid substrates may participate in essential biological responses such as the onset and resolution of inflammation. Some other formulas also contain medium-chain triglycerides (MCT) from coconut oil. Medium-chain triglycerides do not require bile salts for digestion and are rapidly hydrolyzed and well-absorbed, finding an indication in conditions of maldigestion/malabsorption which are still amenable to enteral nutrition. Whether the presence of MCT in enteral tube feeding formulas may confer additional clinical benefit is unclear. The present systematic review was performed in order to verify whether the presence of specific lipidic substrates, namely MCT, in enteral tube feeding formulas is associated to measurable clinical benefits in patients receiving enteral nutrition in different clinical settings, including home enteral nutrition (HEN).

## Methods

Pubmed database was searched (date of search: June–December 2019) for papers with the following search string: (MCT [MeSH Major Topic]) OR Medium chain Triglycerides ([MeSH Major Topic]) AND enteral nutrition ([MeSH Major Topic]).

**Figure 1:**
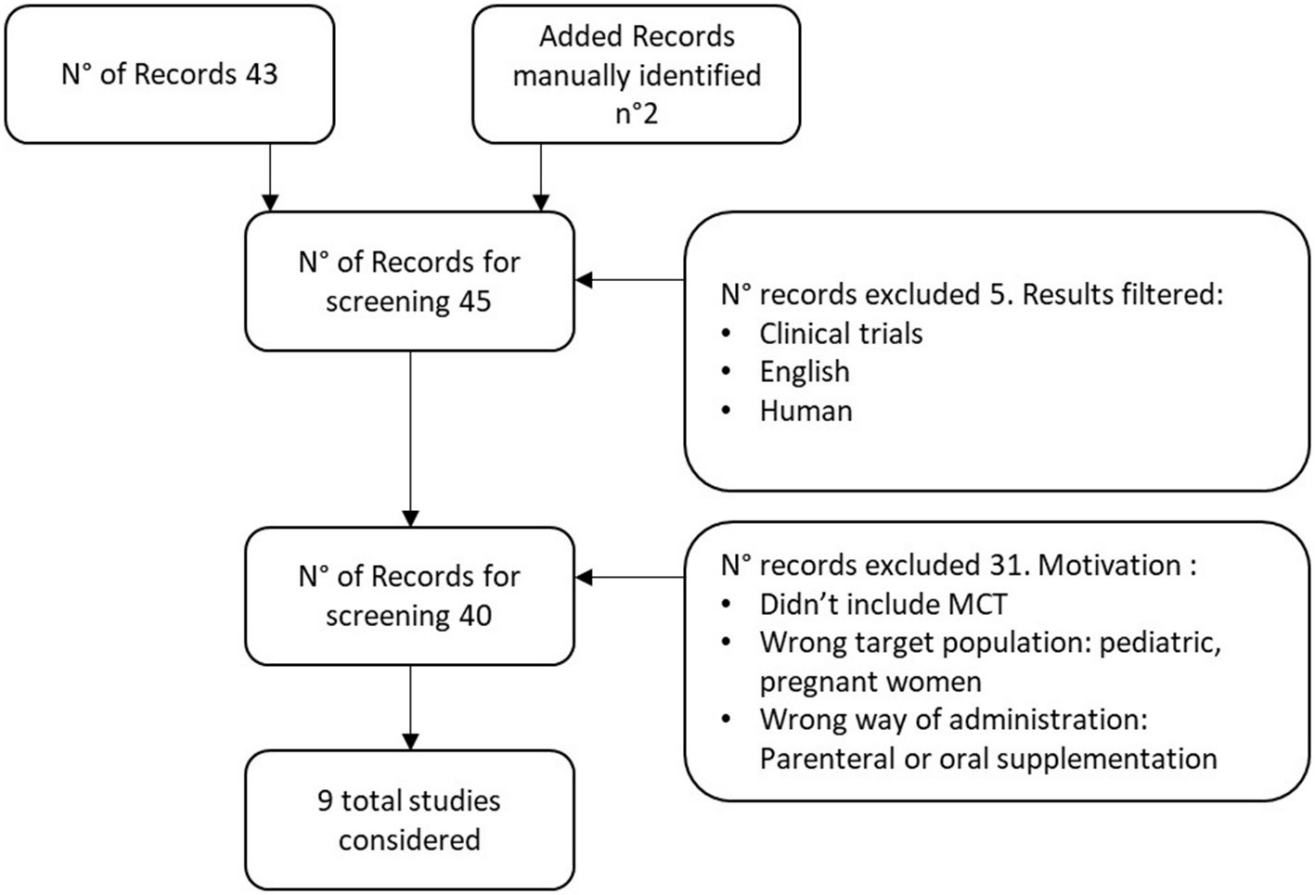
PRISMA Diagram MCT in enteral nutrition. Excluded studies have been reported in Supplementary Table 1, The additional records were identified searching manually in the reference list of those studies deemed relevant.

Identified papers were checked for coherence with the defined inclusion criteria, and the reference list of those deemed relevant was manually searched for further relevant studies, as illustrated in the PRISMA diagrams (Figure 1). The excluded studies and the reasons for exclusions are reported in Supplementary Table 1.

Inclusion criteria were based on the PICOS format (Population, Intervention, Comparison, Outcomes, and Study design). *Population*: publications included adult patients in need of enteral nutritional support either with or whithout specific disease. *Intervention and comparison*. Included papers reported the use of MCT enteral formulas in target population, comparing them to the use of other nutritional support systems. *Outcomes*. Outcomes considered in the evaluation were: frequency and gravity of GI side effects, remission rate, length of stay, or other specific parameters related to the clinical condition of the patients. *Study*. Randomized clinical trials and cohort studies (observational prospective and retrospective studies) published in English in the last 30 years were included.

## Results

The results of the literature search for this systematic review are reported in Table 1.

**Table 1:** Clinical studies included in the systematic review evaluating the effects of MCT-enriched enteral nutrition on clinical outcomes.

	**Patients**	**Study design**	**Type of enteral formula**	**Endpoints**	**Results**
Viall et al. (5)	23 non-surgical hospitalized patients	Randomized clinical trial	MCT-enriched formula Top Up (MCT content: 83.5% of the total lipids); vs. control formula Osmolite (MCT Content: 50% of the total lipids)	GI adverse effect, bowel movements, high gastric residual	No significant differences
Sakurai et al. (6)	37 Crohn's disease patients (CDAI > 150)	Randomized clinical trial	MCT enriched formula Twinline (MCT content 40 g per dose); vs. control formula Elental (MCT content 0 g per dose)	Remission rate at 6 weeks	No significant differences
Wang et al. (7)	229 postoperative patients with gastrointestinal cancer	Randomized clinical trial	MCT and protein enriched enteral formula Nutrison MCT^®^ (MCT content: 2 g /100 ml); vs. control formula Nutrison MF (MCT content: 0 g/100 ml)	Length of stay, diarrhea episodes, postoperative complications.	MCT and protein-enriched EN improves the prealbumin level and shortens the length of hospital stay in patients with gastrointestinal cancer without a high rate of adverse reaction.
Abu Hilal et al. (8)	245 patients that underwent to major pancreatic resection with chyle leak complication.	Cohort study	All patients received the same MCT enriched enteral formula Nutrison MCT^®^ (MCT content: 2 g /100 ml)	MCT tolerability in these patients.	Enriched MCT formulas are safe in this type of patients.
Pan et al. (9)	58 conservative treatment of chylous ascites after abdominal surgery patients	Cohort study	MCT enriched formula vs. MCT diet, vs. TPN.	Tube removal, resumption of oral diet, cost effectiveness.	MCT-containing EN was the best cost-effective nutrition support. The treatment with somatostatin in combination with + MCT-EN is recommended in the conservative treatment of postoperative chylous ascites.
Kawai et al. (10)	30 combined oral and nasal tube feeding anorexia nervosa patients.	Randomized clinical trial	MCT enriched formula PemPal^®^ (MCT content: 2.25 g/100 mL), vs. Isocal RTU^®^ (MCT content: 0.84 g/100 mL), vs. Ensure Liquid^®^ (MCT content: 0 g/100 ml)	Ghrelin and NPY activity	MCT activates ghrelin and increases NPY suggesting a possible treatment.
Jakob et al. (11)	90 ICU patients.	Randomized clinical trial	MCT enriched formula Peptamen AF^®^ (MCT Content: 50% of the total lipids); vs. control formula Isosource^®^ Energy	Diarrhea incidence	Diarrhea incidence was not improved in the test group.
Qiu et al. (12)	144 enterally-fed patients	Randomized clinical trial	Fat Modified Enteral formula TPF FOS^®^ containing MCT + carnitine and taurine (MCT content: 20% of the total lipids); vs. control formula TPF-TP (MCT content: 0%)	EN intake, feeding intolerance (diarrhea, vomiting, gastric retention, and abdominal distension) and outcomes (mechanical ventilator-free days of 28 days, length of ICU stay, length of hospital stay, and in hospital mortality).	The fat-modified enteral formula containing MCT, carnitine, and taurine may improve feeding tolerance in critically ill patients.
Bernini et al. (13)	34, traumatic brain damage (TBI) patients	Cohort study	MCT enriched formula Peptamen AF^®^ (MCT Content: 23 g/1,000 kcal); vs. Promote Fibers Plus^®^ (MCT content: 6.5 g/1,000 kcal)	KB presence	MCT enriched formula did not increase significantly the amount of KB in TBI patients.

*MCT, Medium-chain triglycerides; GI, gastrointestinal; CDAI, Crohn's Disease Activity Index; TPN, total parenteral nutrition; EN, enteral nutrition; NPY, neuropeptide Y; ICU, intensive care unit; EN, enteral nutrition; TBI, traumatic brain injury; KB, ketone bodies*.

In summary, although the use of MCT-containing enteral formulas has been extensive since the early 90's, there is still little evidence about the possible clinical benefits related to their use, when compared to non MCT-containing EN formulas. Few high-quality studies that could clearly demonstrate superiority or inferiority of MCT-containing formulas have been performed since then. Furthermore, as emerged from the systematic review, their broad efficacy on different clinical settings has not been specifically investigated. The available studies include very selected patients populations, making it impossible to draw definite conclusions about the possible benefits of MCT inclusion in enteral formulas for general purposes.

One of the first studies that compared the use of fat MCT modified enteral formulas to standard formulas has been performed by Viall and coll (5). This small double-blind, randomized clinical trial included 23 non-surgical hospitalized patients, considered to be representative of the common adult population requiring enteral nutrition in general practice. Subjects were randomized to receive the study formula (SF: 83,5% MCT + 16.5% LCT) or the control one (CF: 50% MCT + 50% LCT) for a total of 65 days. Although the results were numerically in favor of the SF, no significant differences were observed in the number of bowel movements per day (CF: 1 ± 0.5 vs. SF: 0.6 ± 0,3) or days with high gastric residual (CF: 10/59 vs. SF: 2/59). Similarly, no differences were observed in the rate of gastrointestinal adverse effects as diarrhea (CF: 7/59 vs. SF: 4/59 days) and vomiting (7/59 vs. SF: 1/59 days). The authors themselves acknowledge the small number of patients included as a limitation of the study (5).

In 2002, the effect of an enteral formula with increased MCT (40 g/l) content was tested in Crohn's disease patients, in order to verify if the lipid composition could have an influence on disease activity. More in detail, 37 patients with active Crohn's disease whose Crohn's disease activity index (CDAI) was >150 were enrolled in a prospective, randomized, controlled study that compared an elemental low fat nutrient formula to an MCT-high enteral nutrition. For 6 weeks the patients abstained from food and were administered the assigned nutrient formula by a 24 h/day infusion, through an enteral feeding tube. The rates of disease remission in the 2 groups were determined at 6 weeks to assess short-term therapeutic effects (remission was defined as a reduction of CDAI by at least 40%, or by 100 or more, compared with the baseline). The rate of clinical remission was similar in the two groups (72% in the MCT-enriched formula group vs. 67% in the control group, *p* = ns), suggesting that clinical benefit induced by the short-term treatment with enteral nutrition was not related to the MCT content of the formula. The main limitation is, again, the small sample size (6).

Recently, Bernini et al. investigated the possible use of MCT-enriched enteral nutrition in patients that underwent traumatic brain damage (TBI). Aim of the study was to verify if the continuous enteral feeding with MCT could modulate ketone metabolism, considered to be protective against neuro-induced damages. Thirty-four patients were monitored with cerebral micro- dialysis in order to measure total brain interstitial and circulating levels of ketone bodies (KB) and free fatty acids. Measurements did not show any increase in blood KB, with a modest increase in blood and brain of free medium chain fatty acids, suggesting that ketone metabolism was not influenced by MCT supplementation. This is a small prospective study and the authors specify that the limitations are due to the single-center design and the fact that the caloric targets (7.5 and 15 kcal/kg) were defined arbitrarily.

Another study on MCT-enriched nutrition has involved patients with anorexia nervosa. The aim of Kawai et al. was to investigate if MCT-rich enteral nutrition could influence ghrelin and neuropeptide Y levels in anorexic patients. Ghrelin is a peptide found in the stomach that increases appetite and fat-free mass while suppressing energy expenditure, its active form requires a structural modification by MCTs, necessary passage to exert its physiological action. That's why the authors hypothesized that an increased availability on MCT substrates could increase the levels of active ghrelin and other related mediators (neuropeptide Y). The results of this small study showed significantly higher levels of activated ghrelin in the 30 patients exposed to high doses of MCT. The authors identify as limiting factors the small amount of calories in the patients' diet that make difficult to measure any change in calories intake and that the subjective appetite hasn't been considered (10).

Chyle leaks and abscesses, frequently complicating abdominal surgery procedures (e.g., major pancreatic resection) consist in the pathologic leakage of triglyceride-rich lymphatic fluids into the peritoneal cavity. To find the most appropriate nutritional support in this kind of complicated patients, Pan et al. conducted a retrospective study that examined the nutritional management of 58 patients that incurred in this complication after abdominal surgery. The therapeutic protocol included oral MCT supplementation, total parenteral nutrition, and MCT-containing enteral nutrition. Parameters such as tube removal time, time to resumption of an oral diet, length of hospital stay were better when the treatment with somatostatin was associated to TPN or to MCT + enteral nutrition regime. The article however indicates MCT-containing enteral nutrition as the most cost-effective option. The small size is also this time a possible limitation of the study.

The tolerability of MCT-enriched enteral formulas was retrospectively investigated by Hilal et al. in patients undergoing major pancreatic resection and who developed chyle leaks complications (8). Enterally fed patients did not show increased morbidity or mortality, compared to the control group (parenteral nutrition), and the authors concluded that MCT-enriched formulas may be safely employed in this clinical setting. The main limitation is represented by the retrospective fashion of the study.

The last three studies (7, 11, 12) included in the present systematic review focused more on tolerability of MCT-enriched nutrition vs. a standard MCT-free enteral formula, investigating different clinical outcomes such as the rate of diarrhea episodes, abdominal distension, and length of hospital stay. However these studies are focused on tolerability rather than on the clinical efficacy of the intervention.

More specifically, Jakob et al. started from the observation that one of the most common symptoms in intensive care units is diarrhea, which is associated to discomfort and complications that could increase length of stay and nursing workload. The prospective double-blind study compared the incidence of diarrhea episodes in two groups of patients, respectively, receiving an MCT- and fish oil-enriched formula or a standard enteral formula. No clinical benefit was observed in the treatment group. The relevance of the results is limited by the small number of participants (11).

More positive results were found in the other two studies. Qui et al. enrolled 144 patients in need of enteral nutrition to evaluate nutrient intake, incidence of feeding intolerance, and hard clinical outcomes when patients were treated with a fat-modified enteral formula containing MCT, carnitine and taurine vs. a standard enteral formula. The results of the study pointed out that the fat-modified enteral formulas containing MCT, carnitine, and taurine may improve feeding tolerance in critically ill patients. However, the individual role of the single nutrients was not ascertained, which significantly limits the impact of the study (12).

In 229 patients with cancer of the gastrointestinal tract, Wang et al. showed that a MCT- and protein-enriched enteral formula improves prealbumin level and shortens the length of hospital stay without increasing rate of adverse reactions with respect to patients receiving isocaloric enteral nutrition (7).

## Discussion

Enteral nutrition is indicated whenever oral food intake is either insufficient to meet the nutritional requirements, or if it is contraindicated because of an underlying disease or condition. In both the hospital and home settings, enteral nutrition represents a safe and cost-effective medical treatment (1) to ensure nutrient delivery in a number of clinical conditions in which the gastrointestinal system is accessible and at least partially functional (1), ranging from critical illness (14–16) to neurodegenerative disorders and vegetative state (3). Standard, non-disease specific, enteral nutrition formulas are devised in order to have a nutrient composition which resembles that of a regular oral diet and which can comply with the Recommended Daily Allowance which is defined as “the average daily dietary intake level that is sufficient to meet the nutrient requirements of nearly all (~98 percent) healthy individuals.” Disease-specific formulas have a modified macro-and micronutrient composition which may better satisfy the nutritional and metabolic needs of specific patient categories (e.g., diabetic, neoplastic, critically ill, respiratory patients, etc.). Lipids represent an essential component of enteral formulas. Triglycerides are the main source of fats in a normal dietary regimen; accordingly, artificial formulas contain a balanced share of TG, mainly or exclusively present in the form of corn or soybean oil-derived long-chain triglycerides (LCT). However, other lipid substrates may also be present such as mono-unsaturated fatty acids (MUFA) from safflower and canola oils and medium-chain triglycerides (MCT) from coconut oil (17). As for the oral diet, lipids in enteral formulas represent a major source of energy yielding about 9 kilocalories/g. However, they also provide essential fatty acids, building blocks for fundamental metabolic pathways and “solvent” for lipid soluble vitamins (17). During the last few decades, the vision on the physiological function of lipids has greatly evolved: they have become also functional nutrients that could contribute to the healing process. Lipid modified enteral formulas such as immune-modulating ones (immununutrition is defined as the administration of pharmacologically active substances, such as arginine, glutamine, selenium, ω-3 fatty acids such as EPA and DHA, GLA, nucleotides, and/or antioxidants in efforts to modulate the metabolic response to surgery or stress, by enhancing immune function or by downregulating the inflammatory response) (18) and the replacement/enrichment of LCT with MCT or MUFA are some of the most meaningful examples of this approach.

The addition of MCT to LCT-based enteral formulas has been considered not only to improve the similarity between food and enteral nutrition composition, but also in the attempt to improve tolerance to enteral nutrition in some particular clinical conditions, in particular in patients with impaired intestinal function (s). Long-chain triglycerides require digestion by pancreatic lipase and are mixed with bile salts for absorption, whereas MCT may be absorbed directly across the intestinal mucosa (14). Thanks to their molecular features, MCT do not require the incorporation in chylomicrons and are absorbed directly in the portal circulation, thus facilitating absorption in case of maldigestion, malabsorption or short bowel syndrome. Due to their fast metabolic utilization, MCT promote a ketogenic metabolism (19). Moreover, MCT are relatively resistant to peroxidation (14). Few reviews have comprehensively analyzed the role of enteral nutrition composition in pathological conditions. Ajabnoor et al. performed a systematic review on the fat composition of enteral formulas in Crohn's disease patients. In particular, the composition of the formula was correlated to the remission rate (RR) of the disease. The results of the study showed a positive trend for the ratio of Omega3/Omega 6 fatty acids, while a non-significant positive trend was associated to MCT content. No positive effect was established for other types of fat. Furthermore, no relationship was found between clinical outcomes and other lipid types contained in the formulas (15).

Considering what emerged from this review, it's clear that wider and more statistically significant studies should be carried out to consider the generale use of MCT-enteral containing formulas, in order to answer this not well-investigated topic. If more evidences will be generated it would be possible to reevaluate the administration of this particular formulas in different clinical settings, basing on the evidence based approach, so giving advice to healthcare providers and policy makers. New studies could also impact EN-formula producers and encourage the development of more nutritional adequate products.

Besides the small numbers of patients included in the examined studies, limitation of the systematic review are the use of a single database (Pubmed) for the search and the inclusion of english only paper.

## Conclusions

In conclusion, from the studies included in the present systematic review it appears that, in spite of the strong rationale, there is no clear evidence to recommend inclusion of MCT in both standard and/or disease-specific formulas, or to prefer MCT-enriched over non-MCT-enriched enteral tube feeding. From the studies included in the present systematic review it appears that, in spite of the strong rationale, the addition of MCT to enteral nutrition formulas confers limited benefit, if any, in specific pathological conditions. Moreover, studies evaluating the clinical effects of MCT-enriched enteral nutrition in patients amenable to non-disease specific enteral nutrition are lacking. Evidence exists, however, that enteral nutrition formulas containing MCTs are safe and well-tolerated.

Further, well-designed, adequately powered randomized controlled studies would be needed in order to assess the superiority of MCT- containing enteral formulas over other standard or disease-specific enteral products.

In conclusion, from the studies included in the present systematic review it appears that, in spite of the strong rationale, the addition of MCT to enteral nutrition formulas confers limited benefit, if any, in specific pathological conditions. Studies evaluating the clinical effects of MCT-enriched enteral nutrition in patients amenable to non-disease specific enteral nutrition are lacking.

Based on the available literature, there is no evidence to recommend inclusion of MCT in both standard and/or disease specific formulas and it appears that there are not enough data to support the general use of MCT-containing enteral tube feeding formulas.

Evidence exists, however, that formulas containing MCTs are safe and well-tolerated. Further, well-designed, adequately powered randomized controlled studies would be needed in order to assess the superiority of MCT-containing enteral formulas over other standard or disease specific enteral products.

## Data Availability Statement

The original contributions presented in the study are included in the article/Supplementary Material, further inquiries can be directed to the corresponding authors.

## Author Contributions

MM and LP drafted the manuscript and approved the submitted version.

## Conflict of Interest

The authors declare that the research was conducted in the absence of any commercial or financial relationships that could be construed as a potential conflict of interest.

## Publisher's Note

All claims expressed in this article are solely those of the authors and do not necessarily represent those of their affiliated organizations, or those of the publisher, the editors and the reviewers. Any product that may be evaluated in this article, or claim that may be made by its manufacturer, is not guaranteed or endorsed by the publisher.
