# Supplementary material for: Medium-Chain Triglyceride (MCT) Content of Adult Enteral Tube Feeding Formulas and Clinical Outcomes. A Systematic Review
Source: Front Nutr. 2021 Aug 2;8:697529. doi: 10.3389/fnut.2021.697529 (PMC8364971; doi:10.3389/fnut.2021.697529)
Supplement: Supplementary file 1 [file Data_Sheet_1.PDF]

Supplementary Material

Table 1: Excluded studies and reasons for exclusion.

**Excluded papers**	**Reason for exclusion**
Yildizdas HY, Poyraz B, Atli G, Sertdemir Y, Mert K, Ozlu F, et al. Effects of two different lipid emulsions on antioxidant status, lipid peroxidation and parenteral nutrition- related cholestasis in premature babies, a randomized-controlled study. *Pediatr Neonatol*. (2019) Aug;60(4):359-367. doi:10.1016/j.pedneo.2018.07.012.	Way of administration: parenteral<br>Target population: premature babies
Brumberg HL, Kowalski L, Troxell-Dorgan A, Gettner P, Konstantino M, Poulsen, et al.Randomized trial of enteral protein and energy supplementation in infants less than or equal to 1250 g at birth. *J Perinatol.* (2010) Aug;30(8):517-21. doi: 10.1038/jp.2010.10.	Target population: infants
Klek S, Chambrier C, Cooper SC, Gabe S, Kunecki M, Pironi L, et al. Home parenteral nutrition with an omega-3-fatty-acid-enriched MCT/LCT lipid emulsion in patients with chronic intestinal failure (the HOME study): study protocol for a randomized, controlled, multicenter, international clinical trial. *Trials*. (2019) Dec 30;20(1):808. doi: 10.1186/s13063-019-3994-z.	Way of administration: parenteral
Wang XY, Pan SH, Liu XX, Wu GH, Wang YN, Liu YX, et al. [Effect of high amounts of medium chain triglyceride and protein enteral nutrition on nutritional status in patients after major abdominal operation]. *Zhonghua Wei Chang Wai Ke Za Zhi*. (2007) Jul;10(4):329-32.	Paper not available in English
Arsenault AB, Gunsalus KTW, Laforce-Nesbitt SS, Przystac L, DeAngelis EJ,Hurley ME,et al. Dietary Supplementation With Medium-Chain Triglycerides Reduces Candida Gastrointestinal Colonization in Preterm Infants. *Pediatr Infect Dis J.* (2019) Feb;38(2):164-168. doi: 10.1097/INF.0000000000002042.	Way of administration: dietary supplementation<br>Target population: preterm infants
Symersky T, Vu MK, Frölich M, Biemond I, Masclee AA. The effect of equicaloric medium-chain and long-chain triglycerides on pancreas enzyme secretion. *Clin Physiol Funct Imaging*. (2002) Sep;22(5):307-11. doi: 10.1046/j.1475-097x.2002.00435.x.	Not addressing the clinical questions of the systematic review
Rodriguez M, Funke S, Fink M, Demmelmair H, Turini M, Crozier G, et al.Plasma fatty acids and [13]linoleic acid metabolism in preterm infants fed a formula with medium-chain triglycerides. *J Lipid Res*. (2003) Jan;44(1):41-8. doi:10.1194/jlr.m200218-jlr200. PMID: 12518021.	Way of administration: dietary supplementation<br>Target population: preterm infants
Socha P, Koletzko B, Demmelmair H, Jankowska I, Stajniak A, Bednarska-Makaruk M, et al. J. Short-term effects of parenteral nutrition of cholestatic infants with lipid emulsions based on medium-	Way of administration: parenteral

chain and long-chain triacylglycerols. *Nutrition*. (2007) Feb;23(2):121-6. doi: 10.1016/j.nut.2006.10.009.	Target population: preterm infants
Feinle C, Rades T, Otto B, Fried M. Fat digestion modulates gastrointestinal sensations induced by gastric distention and duodenal lipid in humans. *Gastroenterology*. (2001) Apr;120(5):1100-7. doi: 10.1053/gast.2001.23232.	Not addressing the clinical questions of the systematic review
Wang WP, Yan XL, Ni YF, Guo K, Ke CK, Cheng QS, et al.Effects of lipid emulsions in parenteral nutrition of esophageal cancer surgical patients receiving enteral nutrition: a comparative analysis. *Nutrients*. (2013)Dec 27;6(1):111-23. doi: 10.3390/nu6010111.	Way of administration: parenteral
Larsen BM, Field CJ, Leong AY, Goonewardene LA, Van Aerde JE, Joffe AR, et al. Pretreatment with an intravenous lipid emulsion increases plasma eicosapentanoic acid and downregulates leukotriene b4, procalcitonin, and lymphocyte concentrations after open heart surgery in infants. *JPEN J Parenter Enteral Nutr*. (2015) Feb;39(2):171-9. doi: 10.1177/0148607113505326.	Way of administration: parenteral
Chambrier C, Bannier E, Lauverjat M, Drai J, Bryssine S, Boulétreau P.Replacement of long-chain triglyceride with medium-chain triglyceride/long-chain triglyceride lipid emulsion in patients receiving long-term parenteral nutrition: effects on essential fatty acid status and plasma vitamin K1 levels. *JPEN J Parenter Enteral Nutr*. (2004) Jan-Feb;28(1):7-12. doi: 10.1177/014860710402800107.	Way of administration: parenteral
Wu MH, Wang MY, Yang CY, Kuo ML, Lin MT. Randomized clinical trial of new intravenous lipid (SMOFlipid 20%) versus medium-chain triglycerides/long-chain triglycerides in adult patients undergoing gastrointestinal surgery. JPEN J Parenter Enteral Nutr. 2014 Sep;38(7):800-8. doi: 0.1177/0148607113512869.	Way of administration: parenteral
Wu PY, Edmond J, Morrow JW, Auestad N, Ponder D, Benson J. Gastrointestinal tolerance, fat absorption, plasma ketone and urinary dicarboxylic acid levels in low-birth-weight infants fed different amounts of medium-chain triglycerides in formula. *J Pediatr Gastroenterol Nutr*. (1993) Aug;17(2):145-52. doi:10.1097/00005176-199308000-00004.	Target population: low-birth-weight infants
Cury-Boaventura MF, Torrinhas RS, de Godoy AB, Curi R, Waitzberg DL. Human leukocyte death after a preoperative infusion of medium/long-chain triglyceride and fish oil parenteral emulsions: a randomized study in gastrointestinal cancer patients. *JPEN J Parenter Enteral Nutr*. (2012) Nov;36(6):677-84. doi: 10.1177/0148607111432759. Epub 2012 Jan 26.	Way of administration: parenteral
Yan H, Huang XH, Xiao KJ, Liu XS, Peng YZ, Huang YS, Wang SL. [Effects of medium and long-chain triglyceride on the immune function of burn patients during early postburn stage]. *Zhonghua Shao Shang Za Zhi*. (2003) Aug;19(4):202-5.	Paper not available in english
Shea JC, Bishop MD, Parker EM, Gelrud A, Freedman SD. An enteral therapy containing medium-chain triglycerides and hydrolyzed peptides reduces postprandial pain associated with chronic pancreatitis. *Pancreatology*. (2003);3(1):36-40. doi: 10.1159/000069144.	Way of administration: oral

Puiggròs C, Sánchez J, Chacón P, Sabín P, Roselló J, Bou R, et al. Evolution of lipid profile, liver function, and pattern of plasma fatty acids according to the type of lipid emulsion administered in parenteral nutrition in the early postoperative period after digestive surgery. *JPEN J Parenter Enteral Nutr*. (2009) Sep-Oct;33(5):501-12. doi: 10.1177/0148607109333001	Way of administration: parenteral
Mascioli EA, Randall S, Porter KA, Kater G, Lopes S, Babayan VK, Blackburn GL, Bistrian BR. Thermogenesis from intravenous medium-chain triglycerides. *JPEN J Parenter Enteral Nutr*. (1991) Jan-Feb;15(1):27-31. doi:10.1177/014860719101500127.	Way of administration: parenteral
Senkal M, Geier B, Hannemann M, Deska T, Linseisen J, Wolfram G, et al. Supplementation of omega-3 fatty acids in parenteral nutrition beneficially alters phospholipid fatty acid pattern. *JPEN J Parenter Enteral Nutr*. (2007) Jan-Feb;31(1):12-7. doi: 10.1177/014860710703100112.	Way of administration: parenteral
Sabater J, Masclans JR, Sacanell J, Chacon P, Sabin P, Planas M. Effects on hemodynamics and gas exchange of omega-3 fatty acid-enriched lipid emulsion in acute respiratory distress syndrome (ARDS): a prospective, randomized, double-blind, parallel group study. *Lipids Health Dis*. (2008) Oct 23;7:39. doi:10.1186/1476-511X-7-39.	Wrong way of administration: parenteral
Gelas P, Cotte L, Poitevin-Later F, Pichard C, Leverve X, Barnoud D, et al. Effect of parenteral medium- and long-chain triglycerides on lymphocytes subpopulations and functions in patients with acquired mmunodeficiency syndrome: a prospective study. *JPEN J Parenter Enteral Nutr*. (1998) Mar-Apr;22(2):67-71. doi: 10.1177/014860719802200267. PMID: 9527962.	Way of administration: parenteral
Craig GB, Darnell BE, Weinsier RL, Saag MS, Epps L, Mullins L, Lapidus WI, Ennis DM, Akrabawi SS, Cornwell PE, Sauberlich HE. Decreased fat and nitrogen losses in patients with AIDS receiving medium-chain-triglyceride-enriched formula vs those receiving long-chain-triglyceride-containing formula. *J Am Diet Assoc.* (1997) Jun;97(6):605-11. doi: 10.1016/s0002-8223(97)00155-7.	Way of administration: oral
Li R, Ma J, Yu K, Wang L. Dietary or enteral medium-chain triglyceride usage in a Chinese general hospital. Asia Pac J Clin Nutr. 2015;24(3):387-93. doi: 10.6133/apjcn.2015.24.3.18.	Way of administration: not clearly specified.
Dennison AR, Ball M, Hands LJ, Crowe PJ, Watkins RM, Kettlewell M. Total parental nutrition using conventional and medium chain triglycerides: effect on liver function tests, complement, and nitrogen balance. *JPEN J Parenter Enteral Nutr*. (1988) Jan-Feb;12(1):15-9. doi: 10.1177/014860718801200115.	Way of administration: parenteral<br>Year of publication outside the search period
Ziegler TR. Perioperative nutritional support in patients undergoing hepatectomy for hepatocellular carcinoma. *JPEN J Parenter Enteral Nutr*. (1996) Jan-Feb;20(1):91-2. doi: 10.1177/014860719602000191.	Way of administration: parenteral
Baldermann H, Wicklmayr M, Rett K, Banholzer P, Dietze G, Mehnert H. Changes of hepatic morphology during parenteral nutrition with lipid emulsion containing LCT or MCT/LCT quantified by ultrasound.	Way of administration: parenteral

JPEN J Parenter Enteral Nutr. (1991) Nov-Dec;15(6):601-3. doi: 10.1177/0148607191015006601.	
Diboune M, Férard G, Ingenbleek Y, Bourguignat A, Spielmann D, Scheppler- Roupert C, Tulasne PA, Calon B, Hasselmann M, Sauder P, et al. Soybean oil, blackcurrant seed oil, medium-chain triglycerides, and plasma phospholipid fatty acids of stressed patients. *Nutrition*. (1993) Jul-Aug;9(4):344-9.	Not addressing the clinical questions of the systematic review
Jørgensen L, Trautner F, Engquist A. Mikrobiel kontamination af sondekost [Microbial contamination of tube feeding solutions]. *Ugeskr Laeger*. (1990) Jun 18;152(25):1824-7. Danish. PMID: 2114050.	Paper Not available in english
Calon B, Pottecher T, Frey A, Ravanello J, Otteni JC, Bach AC. Long-chain versus medium and long-chain triglyceride-based fat emulsion in parental nutrition of severe head trauma patients. *Infusionstherapie*. (1990) Oct;17(5):246-8. doi: 10.1159/000222495.	Way of administration: parenteral
Jarnum, S., & Jensen, H. (1966). Medium chain triglycerides (MCT) in the treatment of protein-losing enteropathy and malabsorption syndromes. *Scandinavian journal of gastroenterology*, (1966) 1(4), 306–313. https://doi.org/10.1080/00365521	Year of publication outside the search period
Hill JO, Peters JC, Yang D, Sharp T, Kaler M, Abumrad NN, Greene HL. Thermogenesis in humans during overfeeding with medium-chain triglycerides. *Metabolism.* (1989) Jul;38(7):641-8. doi: 10.1016/0026-0495(89)90101-7.	Way of administration: oral
Stouthard JM, Endert E, Romijn JA, Sauerwein HP. Infusion of long-chain or medium-chain triglycerides inhibits peripheral glucose metabolism in men. *JPEN J Parenter Enteral Nutr.* (1994) Sep-Oct;18(5):436-41. doi: 10.1177/0148607194018005436.	Way of administration: oral
Pontes-Arruda A, Dos Santos MC, Martins LF, González ER, Kliger RG, Maia M, Magnan GB; EPICOS Study Group. Influence of parenteral nutrition delivery system on the development of bloodstream infections in critically ill patients: an international, multicenter, prospective, open-label, controlled study—EPICOS study. *JPEN J Parenter Enteral Nutr*. (2012) Sep;36(5):574-86. doi: 10.1177/0148607111427040.	Way of administration: oral
Rubin M, Harell D, Naor N, Moser A, Wielunsky E, Merlob P, et al.Lipid infusion with different triglyceride cores (long-chain vs medium- chain/long-chain triglycerides): effect on plasma lipids and bilirubin binding in premature infants. *JPEN J Parenter Enteral Nutr*. (1991) Nov-Dec;15(6):642-6. doi: d0.1177/0148607191015006642.	Way of administration: parenteral<br>Target population: premature babies
Sandström R, Hyltander A, Körner U, Lundholm K. Structured triglycerides were well tolerated and induced increased whole body fat oxidation compared with long-chain triglycerides in postoperative patients. *JPEN J Parenter Enteral Nutr.* (1995) Sep-Oct;19(5):381-6. doi: 10.1177/0148607195019005381.	Way of administration: parenteral
